# Melanin Medics: lessons from a community-led organization addressing inequities in medical education

**DOI:** 10.3389/fmed.2026.1864449

**Published:** 2026-06-30

**Authors:** Olamide Oguntimehin, Oyinda Adeniyi, Temidayo Osunronbi, Ayomide Ayorinde, Ellen Nelson-Rowe, Nadia Ibrahim, Khadija Owusu, Ife Akano-Williams, Simisola Sodeinde

**Affiliations:** 1 Melanin Medics, Luton, United Kingdom; 2 School of Allied Health and Social Care, Anglia Ruskin University, Chelmsford, United Kingdom; 3 Lancashire Teaching Hospitals NHS Foundation Trust, Preston, United Kingdom; 4 Mid Yorkshire Teaching NHS Trust, Wakefield, United Kingdom; 5 Guy’s and St Thomas’ NHS Foundation Trust, London, United Kingdom

**Keywords:** allyship, community-led, differential attainment, medical education, mentoring, racial equity, social justice, widening participation

## Abstract

Inequities in health professions education, including differential attainment, racism, and barriers to widening participation, continue to disproportionately affect students from racially-minoritised and underrepresented backgrounds. While there has been a notable rise in institutional responses to racial inequity since 2020, namely the “Black Lives Matter” movement; institutional responses remain individualized, short-term, or deficit-focused. Responsibility to address inequity is often placed on learners within affected groups, rather than structural conditions that produce and sustain inequity. This Perspective article illustrates the development and evolution of Melanin Medics, a student-founded, community-led organization established in response to inequities experienced by Black medical students and doctors. It aims to critique how community-led approaches can contribute meaningfully to social justice and decolonizing efforts in health professions education. Drawing on organizational practice and reflective insight, the article will position Melanin Medics as a case example of how student-led movements can build social capital, affirm professional identities, and challenge inequitable structures. The article concludes by outlining practical implications and key lessons for health professions educators, institutions, and policymakers seeking to advance meaningful action on equity and inclusion.

## Introduction

For many Black medical students and doctors in the United Kingdom (UK), the journey through medicine is defined not only by academic challenge, but also by the compounding experience of isolation, invisibility, and the persistent sense that the profession was not designed with them in mind (1). These experiences are not isolated, but rather reflect the manifestation of the structural inequities deeply embedded within health professions education pertaining to access, attainment and advancement for racially-minoritised groups (2). Efforts to improve diversity and representation within UK medical schools are often described as “widening access” or “widening participation” (3). However, a more diverse medical student population, does not mean equity of experience and can lead to differential attainment. These differences in attainment are evident throughout undergraduate medical education and postgraduate training (4); and adversely impact individuals from racially-minoritised backgrounds (5) who are frequently homogenized within literature as “ethnic minorities” despite their heterogeneity. This risks obscuring key differences in representation and experience resulting in poorly targeted interventions.

While a growing number of institutions have acknowledged both the need to widen access, address systemic racism, and tackle differential attainment, many interventions exist in siloes, remain generalized, designed without meaningful community input and are ultimately lacking (6, 7). The deficit model approach continues to inform literature pertaining to differential attainment, with the emphasis placed on the individual as the “problem” as opposed to the wider system (8). This can have damaging implications on an individual’s sense of professional identity and self-esteem. However, evidence suggests that interventions orientated around belonging, near-peer mentoring, and community of practice can strengthen professional identity formation and self-esteem among underrepresented learners (9). Advancing social justice and equity with medical education and training can be enabled through valuing and centering the lived experience of affected individuals.

In light of the inequities, student-led and community groups have emerged as essential contributors in the landscape of equality, diversity and inclusion work (10). Despite the lived-reality of navigating structurally inequitable environments, these organizations have emerged as communities of practice to create solutions to these very challenges (11), demonstrating the impact of grassroots initiatives and decolonizing the curricula (12). Decolonizing health professions education involves questioning Eurocentric and epistemic perspectives that influence what knowledge is taught, whose experiences are centered, and how structural inequities are reproduced through pedagogical content and norms.

This Perspective article will use Melanin Medics as a case study to reflect on how grassroots work can inform more just, structurally aware education. Melanin Medics, a student-founded, community-led organization established in response to inequities experienced by Black medical students and doctors; with its mission of promoting racial diversity in medicine, widening aspirations and aiding career progression. As a registered charity, Melanin Medics works to promote racial equity in medical education and training in the UK for the benefit of the workforce and patients. The organization does this through educational programmes, social empowerment and the dissemination of valuable resources. Drawing on organisational practice, key design principles and collective reflection, we will examine how community-led movements can contribute to supporting individuals adversely affected by differential attainment and enable structural change, influencing the health professions education environments to meaningfully advance equity.

Throughout this article, we use the term “structural critique” to refer to the process of identifying how current systems, policies and practices produce and sustain racial inequality. This is a necessary precursor to “structural change,” which refers to tangible shifts in institutional policies, curricula and governance. This is consistent with frameworks within the literature that differentiate between promoting broader institutional culture change and embedding structural practices to dismantle racial inequity in health professions education (13).

The authors of this article are core members of Melanin Medics. As black medical students and doctors who have individually navigated the structural inequities described in this article, our perspectives are shaped by lived experience and organisational practice. We consider this positionality to be both a strength, providing insider knowledge and community trust, and a limitation; recognizing that this article does not constitute an independent evaluation of our work.

### Origins, ethos and context of Melanin Medics

Melanin Medics was founded in 2017 by a first year medical student at a UK medical school, who recognized the need for greater representation and visibility of Black medical students and doctors (1). Having navigated the systemic barriers to medical school entry, coming from an underrepresented and lower socio-economic background, she realized the value of mentoring and role modeling. This experience underpinned the importance of near-peer mentoring, communities of practice and visibility of role models in navigating structurally inequitable pathways into medicine and professional identity formation (14). When she started medical school and learned of the experiences of her Black peers, it was then that she realized that her experience was not isolated but reflected well-established patterns of structural exclusion in medical education.

In its earliest form, Melanin Medics began as a blog, an online platform increasing the visibility and accessibility of resources to support aspiring medical students and medical students fostering a sense of shared experience and community among Black aspiring doctors. The visibility of the initiative attracted 10000 views within the first 6 months; a response that evidenced significant unmet need for connection amongst Black medical students and early career doctors. Melanin Medics emerged during a period where widening access initiatives were predominantly orientated around socio-economic background (15), with limited attention to the racialized aspects of belonging.

Over time, Melanin Medics evolved into a national organization, which has grown to become the largest intergenerational network for Black medical students and doctors in the UK. The organization has worked to deliver formal mentoring and career development programmes; create culturally relevant resources and foster community through networking events. This growth was sustained through the voluntary labor of Black medical students and doctors.

Through the educational programmes, the organization has gained institutional recognition and served as a case study for initiatives tackling differential attainment in medical education and training by the General Medical Council (16). Demonstrating how student-led advocacy can change systems, Melanin Medics has influenced policy through the development of the British Medical Association (BMA) Racial Harassment Charter for Medical Schools (17). As part of efforts to challenge Eurocentric curricula, Melanin Medics continue to deliver CPD-accredited “Allyship and Advocacy” training across health professions education at both an undergraduate and postgraduate level. The training workshops place emphasis on anti-racism and the implications of racial inequity and social injustice on patients and the wider healthcare workforce. These workshops have been delivered to over 2000 participants at institutions and healthcare organizations across the United Kingdom.

This trajectory, from grassroots advocacy to structured national organization demonstrates how student-led action, rooted in community need and structural critique, can scale into structural change.

### Designing community-led mentoring and career development

Traditional models of support often fail to account for the structural barriers, cultural contexts and lived experiences that shape the educational trajectories of Black medical students and doctors. In response, Melanin Medics developed a community-led model of mentoring and career development programmes. Over 9 years, the organization has delivered 20 cohorts across seven bespoke mentoring and career development programmes to 558 participants through a range of formats varying in duration, design and delivery.

The following principles emerged iteratively through 9 years of programme development, delivery and community feedback. They are not a prescriptive framework, but rather a summation of organizational insights grounded in practice.

**Principle 1: Lived Experience as Expertise**

Each of our seven programmes were shaped by Black medical students and doctors who had first-hand experience of navigating the journey from medical school applications to accessing specialty training. This positions lived experience as a form of expertise; this methodological choice had direct implications for programme relevance, cultural credibility and community trust. It actively challenges deficit-oriented approaches with an asset-based approach that values what communities already hold, contextual knowledge and cultural insight (18).

**Principle 2: Near-Peer, Race-Concordant Mentoring**

Traditional hierarchical mentoring models may privilege established networks, reducing accessibility and relatability for racially minoritised learners (19). In contrast, Melanin Medics’ mentoring model deliberately centers shared lived experience and cultural affinity. Each mentee was paired with a mentor of Black heritage, increasing the cultural specificity and contextual relevance of the guidance offered. Within the Envision Med Programme, aspiring medical students are paired with current medical students, whereas final-year medical students in the Enrichment Programme are matched with early-career resident doctors (20, 21). This intergenerational structure is a deliberate response to the absence of visible and accessible role models for Black learners within medicine (18). This facilitates credible near-peer, role-modeling grounded in shared structural experience rather than abstract aspiration. Evidence suggests that race concordance is among the most valued dimensions of mentoring relationships for underrepresented learners (22).

**Principle 3: Community as a Site of Knowledge and Connection**

Effective community-led mentoring extends beyond one-to-one relationships. Community events including speaker sessions, panel discussions, and networking opportunities enabled participants to engage with professionals across a range of backgrounds and ethnicities, intentionally mirroring the diversity of the real clinical working environment and encouraging the building of professional relationships across differences. This reflects Melanin Medics’ broader commitment to allyship; recognizing that structural change requires partnerships, not only within communities. This approach also treats the community itself as a productive site of knowledge, functioning to actively distribute social capital within a marginalized group. Internal programme feedback has consistently shown a strengthened sense of belonging and enhanced clarity in career decision-making; outcomes that reflect both the mitigation of exclusion and the affirmation of professional identity (23).

These principles are not unique to Melanin Medics. They hold demonstrable value and offer a transferable framework for community-led design that institutions and organizations looking to advance equity in health professions education may wish to consider and adapt.

### Allyship, advocacy and partnerships with institutions

Alongside direct support for learners, Melanin Medics has developed a complementary stream of work focused on allyship, advocacy and institutional partnership; recognizing that individual support and structural critique must operate in tandem.

The burden of addressing racial inequity in health professions education often falls disproportionately on those most harmed by it; a reality that Melanin Medics has navigated. The evidence suggests that institutional commitments to racial equity have not always translated into meaningful change, especially as learners with marginalized identities continue to report discrimination. These experiences range from microaggressions, social exclusion, tokenism, diversity optics, to systemic inequities embedded in everyday cultural norms (24). Even where institutions have introduced and embedded diversity initiatives or interventions, the gap between stated commitment to culture change and lived experience persists (25). Moreover, discrimination also presents simultaneously across individual, institutional, and societal levels (26). Within medical education, this is reproduced through curriculum design, differential attainment and hiring decisions. Therefore, community-focused responses and solutions that engage across these levels are pivotal.

Melanin Medics has built its approach around this lens. The “Allyship and Advocacy” workshops focus on raising awareness and equipping learners, through proactive bystander training, with the knowledge and practical tools to recognize, name, and address discriminatory practices. The workshops also foster psychologically safe environments where learners can develop a critical understanding of inequitable systemic structures and how to translate that understanding into action. This approach reflects the substantive theory of change, where individual behavior is shaped by institutional conditions, and that shifting those conditions requires people who are willing and able to act within their own spheres of influence (27).

Communities of practice that interact and learn together regularly generate collective knowledge and embed change beyond what individual action achieves alone (28). This principle also underpins Melanin Medics’ approach to institutional partnership. The organization brings together people with lived experiences of racial inequity within healthcare and healthcare education, allowing shared knowledge to develop across time and contexts, and translating that knowledge into advocacy, policy influence and community growth.

The organization’s contribution to the BMA Racial Harassment Charter for Medical Schools is evidence of what this looks like in practice - grassroots expertise and community knowledge, alongside institutional co-creation that scaffolds for formal systemic efforts to promote cultural humility at national levels (29). Melanin Medic’s cross-sector reach extends across higher education, the NHS, and the private sector.

Research on community-academic partnerships warns against institutions drawing on the credibility and reach of community-led organizations without reciprocal strategic commitment (30). Melanin Medics’ model resists this by being grounded in transparent resource and responsibility sharing. For equity in medical education to move beyond rhetoric, institutions must engage community- led organizations as partners in changing the structures they control and take accountability when they do not.

## Discussion

Historically, the role of “affinity groups - groups formed around a shared identity” or “ethnicity-specific associations” has been critiqued based on the thought that groups formed on the basis of ethnicity risk marginalizing individuals further and inadvertently promote segregation (31). While this critique may be reflected less within more recent literature, it is important to explore why organizations like Melanin Medics are still necessary today. The ethos of Melanin Medics is to “support the individual and influence their environment” (32). This two-pronged approach emphasizes that Melanin Medics is not a separatist organization, but rather, prioritizes driving structural change by working across and with institutions, influencing national policy and shaping medical education and training through the delivery of workshops and providing psychologically safe, brave spaces.

Secondly, it is important to recognize that the organization has designed necessary interventions to champion social justice in health professions education in response to structural conditions propagated by the system at large. Thirdly, more-recent evidence has highlighted the limitations of integration-only approaches and how this does not always translate into a sense of belonging and meaningful inclusion within the wider medical community (1). In this context, Melanin Medic’s work embodies decolonizing principles by giving Black medical students and doctors a meaningful role in shaping educational priorities and challenging structural hierarchies that have historically excluded their perspectives.

Reflecting on our organizational journey, a number of lessons emerge relevant to health professions educators, institutions, and policymakers dedicated to advancing social justice in health professions education.

### Key lessons

**Lesson 1: Community knowledge as expertise**

Melanin Medics has demonstrated the value of student-led action, when grounded in lived experience of structural inequity cannot be replicated by institutional researchers working from outside of the community in the absence of co-creation. Community organizations hold deep, contextualized knowledge of the structural barriers racially-minoritised groups face, which must be recognized and valued.

**Lesson 2: Support and structural critique are not competing functions**

Community organizations hold a unique position in maintaining grassroots functions and proximity to members, while engaging with the wider system in order to advocate for change. Organizations, like Melanin Medics have demonstrated the ability to simultaneously support individuals and challenge the conditions that make that support necessary. Structural change is necessarily slow. In dismantling existing inequitable structures, community organizations must therefore continue to provide direct support in parallel, rather than being asked to wait for structural change that has not yet materialized.

**Lesson 3: Near-peer, race-concordant mentoring as a structural response to inequity**

Evidence has highlighted that racial discrimination within health professions education is not simply an uncomfortable experience, but poses a direct threat to professional identity formation and career progression (33). Organizational sensitivities around discussions on race remain; this can have a damaging impact on those affected by racial discrimination within their institutions. Near-peer mentoring has been found to improve self-efficacy, belonging and professional goal orientation among underrepresented learners (34). The Melanin Medics intergenerational network model, intentionally centers race-concordant pairing across the full career trajectory from aspiring medical student, to practicing doctor, achieving cultural specificity and scale that institutional programmes have rarely replicated.

### Calls to action for practice and policy

For educators, we call for the dismantling of deficit framing in the conceptualization, design and delivery of initiatives that aim to address inequities in healthcare professions education. In addition, we call for a move toward asset-based approaches to medical education, shifting from a focus on what is missing, to placing emphasis on strengths within learners from diverse backgrounds (18).

For institutions, we call for the prioritization of long-term, non-extractive partnerships with community-led organizations. Structural change requires long-term commitment, iterative learning and a willingness to engage in the uncomfortable work of institution self-examination. The work of organizations like Melanin Medics is often positioned on the margins of institutional equity work; acknowledged and celebrated, yet not embedded into curricula, clinical training and institutional strategy. Institutions must move beyond awareness days and one-off workshops, toward sustained, resourced and accountable community partnership.

For policymakers, we call for dedicated funding of evaluation infrastructure for community-led programmes. The absence of robust outcome data for organizations like Melanin Medics is not a reflection of organizational failure but rather systemic under-resourcing. Funders should provide resources for independent evaluation rather than placing the onus on volunteer-led organizations to bear this cost themselves.

### Future directions and limitations

Future research may wish to explore longitudinal evaluation frameworks for community-led programmes in health professions education to strengthen the evidence base for interventions addressing inequity. In addition, there is a need for further intersectional analysis disaggregating outcomes by gender, class, disability and place of primary medical qualification within the Black medical student and doctor population.

We acknowledge the limitations of this article. Our reflections draw mainly on our own organizational practice and experience, and do not form a systematic evaluation of our interventions and impact. The absence of consistent formal programme evaluation reflects the reality of individuals within the organization simultaneously navigating the very inequities they seek to address with access to limited resources. We recognize that the lessons drawn from our experience may not be internationally applicable, particularly in regions like the Global South. Further consideration of how geographical context plays a role in health professions education inequities, demographics and professional identity formation is necessary.

In conclusion, the journey of Melanin Medics demonstrates both the power and limitations of student-led and community-led groups. The work mentoring, connecting and advocating for Black medical students and doctors across the UK, demonstrates what works. In order to make irreversible progress in tackling inequities in health professions education, we must progress beyond short-term initiatives toward structural change. Educators, institutions and policymakers are encouraged to work collaboratively with community groups and those most affected by inequity to produce lasting change in how we educate and support future health professionals.

## Data Availability

The original contributions presented in this study are included in this article/supplementary material, further inquiries can be directed to the corresponding author.
